# Prevalence and Associated Factors of Low-Level Viremia in Chronic Hepatitis B Patients After Long-Term Therapy with Nucleos(t)ide Analogs

**DOI:** 10.5152/tjg.2023.21978

**Published:** 2023-01-01

**Authors:** Jiajia Han, Yifei Guo, Xueyun Zhang, Yao Zhang, Jian Sun, Jingjing He, Richeng Mao, Yuxian Huang, Jiming Zhang

**Affiliations:** 1 Department of Infectious Diseases, Shanghai Key Laboratory of Infectious Diseases and Biosafety Emergency Response, National Medical Center for Infectious Diseases, Huashan Hospital, Fudan University, Shanghai, China

**Keywords:** Antiviral agents, chronic, hepatitis B, risk factors, viremia

## Abstract

**Background:**

Low-level viremia is usually defined as a detectable but lower than 2000 IU/mL hepatitis B virus DNA level after 12 months or longer duration of antiviral therapy in chronic hepatitis B patients. In this study, we aimed to clarify the factors associated with low-level viremia in patients during long-term monotherapy with tenofovir disoproxil fumarate or entecavir.

**Methods:**

Chronic hepatitis B patients having received entecavir or tenofovir disoproxil fumarate treatment for 12 months or more were enrolled from October 2019 to October 2021 at a tertiary hospital in Shanghai, China. In accordance with their hepatitis B virus DNA levels, chronic hepatitis B patients were grouped into 3 categories, hepatitis B virus DNA > 2000 IU/mL, low-level viremia, and complete virological response (hepatitis B virus DNA < 10 IU/mL). Compared with complete virological response patients, factors related to low-level viremia were evaluated.

**Results:**

This study enrolled a total of 160 chronic hepatitis B patients, whose duration of treatment ranged from 12 to 144 months. In total, 107 patients achieved complete virological response, 51 showed low-level viremia, and 2 showed hepatitis B virus DNA > 2000 IU/mL. After multivariate logistic regression analysis, hepatitis e antigen-positivity (odds ratio = 6.479, 95% CI: 2.480-16.922, *P* = .000), entecavir treatment (odds ratio = 4.742, 95% CI: 1.855-12.118, *P* = .001), and duration of therapy (odds ratio = 0.168, 95% CI: 0.072-0.388, *P* = .000) were independently associated with low-level viremia.

**Conclusion:**

Having received long-term antiviral treatment, low-level viremia still occurred in 31.9% of patients. Longer duration of therapy was a protective factor, and HBeAg-positivity and entecavir treatment were risk factors for low-level viremia.

**Main Points**

- Low-level viremia (LLV) still occurs in 31.9% of chronic hepatitis B patients who have received more than 12 months of antiviral treatment.
- HBeAg-positivity and entecavir (ETV) treatment are risk factors for LLV, and longer duration of therapy (> 36 months) is a protective factor.
- Tenofovir disoproxil fumarate may perform better than ETV in reducing the occurrence of LLV.

## Introduction

Chronic hepatitis B virus (CHB) infection remains a significant global disease burden. In 2019, 296 million people were living with hepatitis B virus (HBV) infection, and each year, about 1.5 million people are newly infected worldwide.^1^ About 0.8 million people died due to HBV-related diseases in 2019.^1^ Serum HBV DNA level is an important factor that affects prognosis^2,3^; therefore, in CHB patients, the primary goal of treatment is long-term suppression of HBV replication.^4^ Entecavir (ETV), tenofovir disoproxil fumarate (TDF), and tenofovir alafenamide fumarate (TAF) have been commonly used as first-line antiviral drugs for chronic HBV infection because of their potent antiviral ability and low resistance advantages.^5-7^ Moreover, pathological changes in the liver can be improved, and the occurrence of hepatocellular carcinoma (HCC) can be reduced after long-term antiviral therapy.^4,8^ After a 48-week antiviral therapy, 50%-80% of patients can achieve complete virological response (CVR) (serum HBV DNA levels below the lower detection limit, usually 10 IU/mL, 12 IU/mL, 15 IU/mL, or 20 IU/mL).^5,9^ However, there are still 10%-40% of patients who have persistent or occasional low-level viremia (LLV) (serum HBV DNA levels above the lower detection limit but below 2000 IU/mL)^10^ and 10% of patients with above 2000 IU/mL serum HBV DNA levels.^11^ Recently, with the increasing sensitivity of HBV DNA detection methods, the occurrence of LLV has attracted increasing attention. It has been found that LLV is a risk factor for drug resistance development,^12,13^ the progress of cirrhosis,^14^ hepatic carcinogenesis,^11,15-18^ and lower survival rate of HCC patients.^19^ However, the lower detection limit of HBV DNA tests in most areas of China, which varies from 50 IU/mL to 1000 IU/mL, is not consistent with guideline recommendations, which are 10 IU/mL, 12 IU/mL, or 15 IU/mL.^5-7,20^ Therefore, data on the incidence of LLV in China are relatively insufficient. Furthermore, hepatitis e antigen (HBeAg)-positivity and high baseline HBV DNA levels have been generally regarded as risk factors of LLV,^10,21^ but the follow-up in most studies has been relatively short. In our study, we investigated the prevalence rate of LLV in a tertiary hospital in Shanghai, China. We explored the associated factors of LLV in patients having received long-term antiviral therapy (varying from 12 months to 144 months). The clinical significance of this study is to arouse the attention of clinicians to this specific group of patients who are still in the LLV state after long-term antiviral treatment.

## Materials and Methods

### Patients

**Figure 1.**
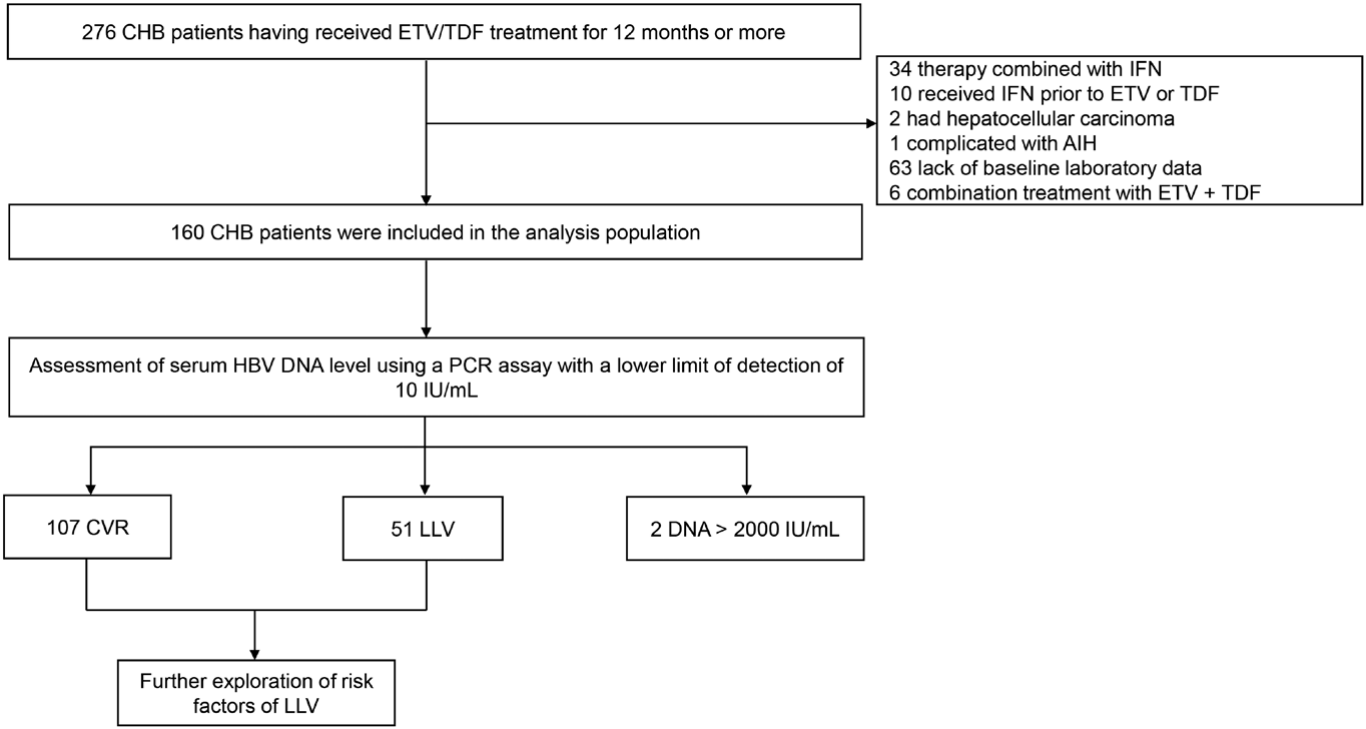
Flowchart of study design. Totally 276 CHB patients who received TDF/ETV treatment for more than 12 months and visited a tertiary hospital in Shanghai, China, between October 2019 and October 2021 were recruited; 116 were excluded. The remaining 160 patients’ serum HBV DNA levels were assessed by a commercially available polymerase chain reaction assay with a lower limit of detection of 10 IU/mL (Abbott, Des Plaines, Ill, USA). 107 patients achieved complete virological response (CVR) with HBV DNA less than 10 IU/mL, 51 patients in a low-level viremia (LLV) state with HBV DNA between 10 IU/mL and 2000 IU/mL, and 2 patients HBV DNA more than 2000 IU/mL. The patients with CVR and LLV were further studied to explore the risk factors of LLV. CHB, chronic hepatitis B; ETV, entecavir; TDF, tenofovir disoproxil fumarate; CVR, complete virological response; LLV, low-level viremia; IFN, interferon; AIH, autoimmune hepatitis.

Totally, 276 CHB patients at a tertiary hospital in Shanghai, China, from October 2019 to October 2021 who had received ETV (0.5 mg/day) or TDF (300 mg/day) treatment (naïve or experienced) for no less than 12 months were screened in this study; 116 were excluded (Figure 1). Patients who met the following criteria were included: (1) patients who had received TDF or ETV for no less than 12 months, (2) patients who had reached the age of 18, (3) patients whose serum hepatitis B surface antigen remained positive for 6 months or more before antiviral therapy, and (4) patients who had a good medication adherence (adherence rate over 80%). The exclusion criteria were (1) lack of baseline clinical data; (2) coinfection with other viral hepatitis or human immunodeficiency virus; (3) co-existence of drug-induced liver disease, genetic liver diseases, autoimmune hepatitis, and other serious diseases; (4) history of HCC or other malignancy; (5) treatment in combination with other antiviral agents, such as ETV + TDF, nucleos(t)ide analogs + interferon; and (6) a history of interferon treatment. This study was approved by the local ethical committee and was carried out according to the Helsinki Declaration. Each patient has signed an informed consent, in which the use of medical records for the publication of scientific research was clearly stated.

### Data Collection

Patients’ demographic data and clinical characteristics at baseline and at the time point of identifying LLV by a more sensitive HBV DNA test (lower detection limit, 10 IU/mL) were collected through medical records, including gender, age, body mass index, alcohol intake, family history of hepatitis B/HCC, duration of disease since the first diagnosis, type of drug, history of antiviral therapy, duration of treatment, liver cirrhosis, fatty liver disease, comorbidities (diabetes mellitus, hypertension), and platelet count, hepatitis B surface antigen (HBsAg) level, alanine aminotransferase (ALT), HBeAg status, HBV DNA, albumin globulin (ALB), aspartate aminotransferase (AST), total bilirubin (TB), γ-glutamyltranspeptidase (GGT), creatinine, alpha-fetoprotein (AFP), prothrombin time, hemoglobin (Hb), and the count of white blood cells (WBC), neutrophil, and lymphocyte. Adherence to ETV or TDF was assessed by inquiry of patients and review of pharmacy records. The adherence rate was calculated as follows: 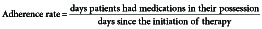 Alcohol intake of more than 20 g per day was recorded. Liver cirrhosis and fatty liver disease were diagnosed according to the results of imaging examination of the liver (abdominal ultrasonography/magnetic resonance imaging/computed tomography) and liver biopsy (if available).

### Laboratory Measurements

An automatic blood cell analyzer (Coulter LH 750, Beckman, Fullerton, Calif, USA) was used to detect complete blood counts. Serum HBsAg, hepatitis B core antibody (anti-HBc), and HBeAg levels were assessed using the Abbott ARCHITECT® i2000SR platform found on a chemiluminescent microparticle immunoassay. The detection range of HBsAg was 0.05-250.0 IU/mL, and sera with HBsAg levels above 250.0 IU/mL were subsequently serially diluted at 1 : 500. A real-time polymerase chain reaction assay (DAAN Diagnostics, Guangzhou, China) was used to measure baseline serum HBV DNA with a 500 IU/mL lower detection limit. The last test of HBV DNA for distinguishing LLV and CVR was measured by Abbott m2000 RealTime assays (Abbott, Des Plaines, Ill, USA) with a lower detection limit of 10 IU/mL. Alanine aminotransferase, AST, TB, ALB, GGT, and creatinine levels were tested by an automatic biochemical analyzer (Hitachi 7600P, Hitachi, Japan).

### Definitions

Complete virological response was regarded as a state where serum HBV DNA level was below 10 IU/mL after no less than 12 months of ETV/TDF therapy. Low-level viremia was usually defined as HBV DNA < 2000 IU/mL but > 10 IU/mL after no less than 12 months of ETV/TDF therapy.^11^ The baseline was the time point before treatment if patients were treatment-naïve or before transformation to ETV/TDF if patients were treatment-experienced.

### Statistical Analysis

The analyses were performed using Statistical Package for the Social Sciences software version 23 (IBM Corp.; Armonk, NY, USA) and GraphPad 8.0 (GraphPad Software, San Diego, Calif, USA). Continuous variables were described using mean ± standard deviation or medians (interquartile ranges), and categorical variables were expressed as percentages (frequencies). The Student’s *t*-test for normally distributed data and Mann–Whitney *U* test for non-normal distribution data were used to analyze continuous variables. Chi-squared test or Fisher’s test was used for comparing categorical variables as appropriate. To identify the independent factors significantly associated with LLV, the multivariate logistic regression model was used, and candidate variables whose *P-value* was <.1 on univariate analysis were put into the regression analysis. In order to better compare the incidence of LLV in ETV and TDF groups, propensity score matching (PSM) on baseline HBeAg state, duration of treatment, history of antiviral therapy, HBV DNA level was applied with pairs being matched within a caliper of 0.05 of the standard deviation (SD) of the logit of the propensity score. Two-tailed *P* values were used, and *P* < .05 was set as the significance level.

## Results

### Patients’ Characteristics

**Figure 2.**
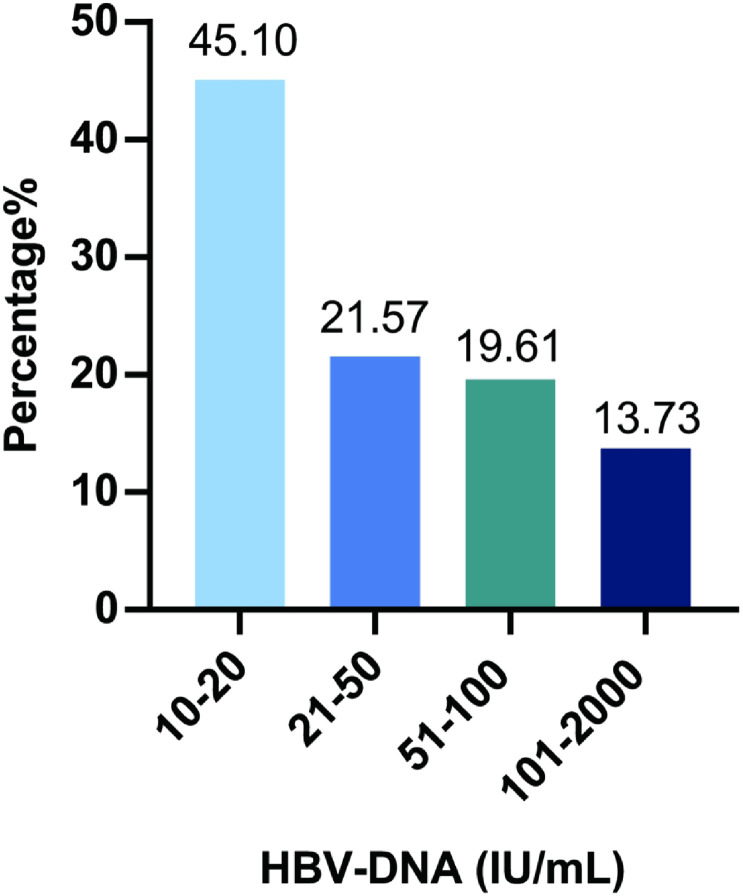
HBV DNA level distribution in CHB patients with LLV (n = 51). DNA was divided into 4 groups (10-20 IU/mL, 21-50 IU/mL, 51-100 IU/mL, 101-2000 IU/mL). HBV, hepatitis B virus; CHB, chronic hepatitis B; LLV, low-level viremia.

This study included 160 patients having received 12 months or longer duration of therapy, and their plasma was examined with more sensitive HBV DNA tests (lower detection limit, 10 IU/mL). Figure 1 shows the flow diagram of the study population. The median duration of treatment was 36 months. Based on the HBV DNA levels, 107 patients (66.88%) achieved CVR, 51 (31.88%) achieved the LLV, and 2 patients (1.25%) had HBV DNA > 2000 IU/mL; the former 2 categories were further analyzed to explore the risk factors of LLV. Of the 158 subjects, 105 (66.46%) were HbeAg-positive; 109 (68.99%) received EVT, 49 (31.01%) received TDF treatment, and 126 (79.75%) were treatment-naïve. Hepatitis B virus DNA levels in the LLV group (n = 51) were between 10 IU/mL and 768 IU/mL (Figure 2), with a median of 23 IU/mL. Table 1 shows the clinical characteristics of CVR and LLV patients. The age distribution (*P* = .958) and gender (*P* = .663) were similar between the patients with CVR and LLV. Compared with LLV group, patients with CVR had lower serum HBV DNA (5.7 vs. 7.0 log_10_ IU/mL, *P* = .013), HBsAg (3.41 log_10_ vs. 3.79 log_10_ IU/mL, *P* = .001), GGT (20 vs. 34 U/L , *P* = .004), AFP levels (2.38 vs. 3.07 ng/mL, *P* = .018), less percentage of patients with positive-HBeAg (57.9% vs. 84.3%, *P* = .001), treated with ETV (63.6% vs. 80.4%, *P* = .032), a higher level of albumin (47.0 vs. 45.0 g/L, *P* = .042), and longer duration of treatment (36.0 vs. 26.4 months, *P* = .020).

#### Associated Factors of Low-Level Viremia Using Multivariate Logistic Regression Analyses

To determine the associated factors of LLV, baseline HBeAg state, the levels of HBV DNA, HBsAg, plasma albumin, GGT, duration of treatment, types of drugs, and history of antiviral therapy related to LLV were further analyzed by a multivariate logistic regression model. The results revealed that ETV treatment (odds ratio (OR) = 4.742, 95% CI: 1.855-12.118, *P* = .001) and HBeAg-positivity (OR = 6.479, 95% CI: 2.480-16.922, *P* = .000) were independent risk factors for the outcome of LLV, and longer duration of treatment (>36 months) was a protective factor (OR = 0.168, 95% CI: 0.072-0.388, *P* = .000) (Table 2).

#### Propensity Score Matching for Better Comparing the Incidence of Low-Level Viremia Between Entecavir and Tenofovir Disoproxil Fumarate Treatment Groups

Totally 36 pairs of CHB patients were identified after matching for baseline HBeAg state, duration of treatment, history of antiviral therapy, and HBV DNA level. After PSM, baseline important clinical indicators between 2 groups indicated good balance (Table 3). The incidence of LLV in the ETV group was significantly higher than that in the TDF group (36.1% vs. 13.9%, *P* = .029).

#### Cross-sectional Comparison Between Low-Level Viremia and Complete Virological Response Groups at the Time Point of Identifying Low-Level Viremia

**Figure 3.**
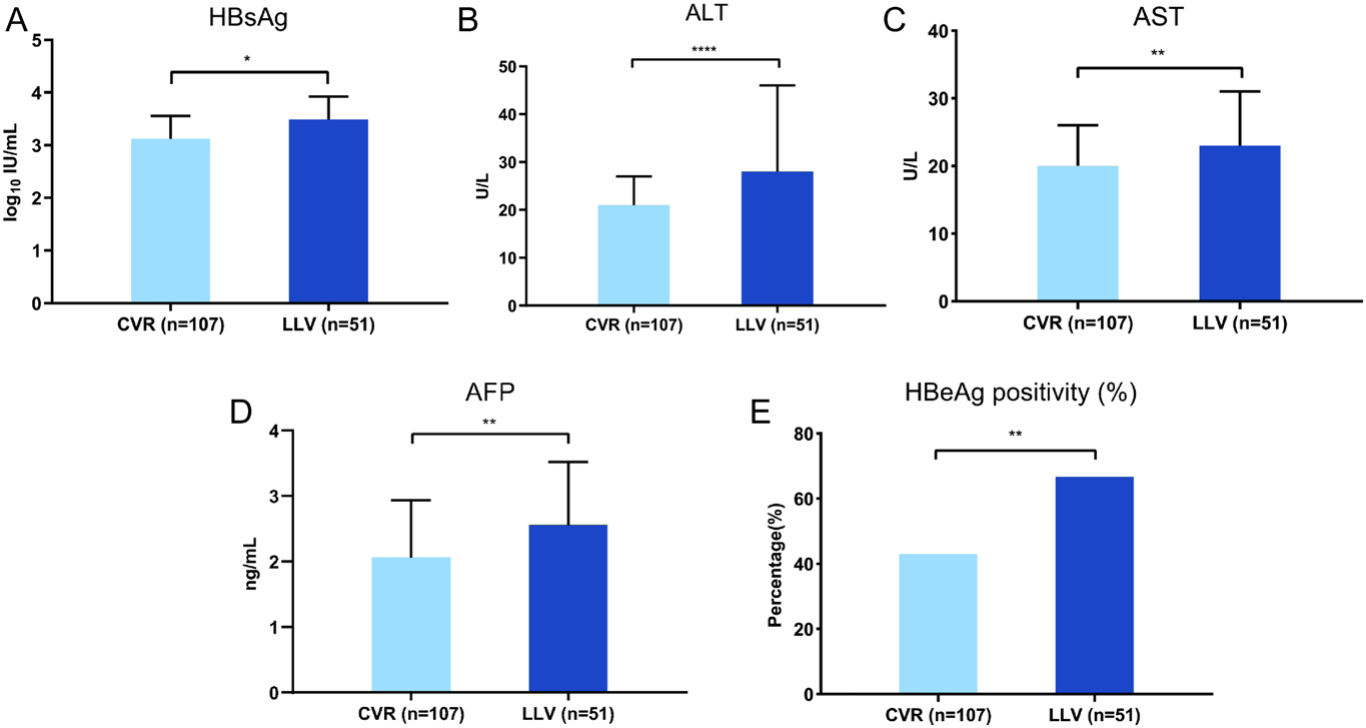
Comparison of HBsAg, ALT, AST and AFP levels and percentage of patients with HBeAg-positivity between CVR and LLV groups at the time point highly sensitive HBV DNA tests (LDL, 10 IU/mL) were performed. ^*^P < .05; ^**^P < .01; ^****^P < .0001. CVR, complete virological response; LLV, low-level viremia; HBV, hepatitis B virus; LDL, lower detection limit; HBsAg, hepatitis B surface antigen; ALT, alanine aminotransferase; AST, aspartate aminotransferase; AFP, alpha fetoprotein; HBeAg, hepatitis B e antigen.

Compared with CVR groups, patients with LLV had a detectable HBV DNA with a higher serum ALT (28.0 vs. 21.0 U/L), AST (23.0 vs. 20.0 U/L), AFP (2.56 vs. 2.06 ng/mL), and HBsAg (3.49 vs. 3.12 log_10_ IU/mL) levels and a higher percentage of HBeAg-positivity (66.7% vs. 43.0%) (Figure 3 and Table 4).

## Discussion

This study found that LLV was up to 31.9% in long-term ETV/TDF treatment of CHB patients when the lower detection limit was 10 IU/mL, which was consistent with a previous study.^22^ Baseline HBeAg-positivity and high HBV DNA are widely recognized as risk factors for LLV.^23-25^ We also found HBeAg-positivity at baseline as an independent risk factor, but HBV DNA was excluded after multivariate logistic regression. This is most likely because our study’s upper detection limit of HBV DNA tests was 10^7^, which narrowed the gap in DNA levels between the LLV and CVR groups. In our study, TDF seems superior to ETV in virological response. The difference in virological response between ETV and TDF was controversial in previous studies, and some reached similar conclusions to ours.^25-27^ We also found that as the duration of therapy was prolonged, the occurrence of LLV decreased, and patients with a treatment duration of less than 36 months had a higher incidence of LLV than those treated for more than 36 months. Based on the above results, prolonging the duration of treatment or changing to TDF is reasonable to deal with LLV. There is no unanimous conclusion on how to deal with LLV. The guidance of the American Association for the Study of Liver Diseases recommended continuing monotherapy.^5^ The European Association for the Study of Liver recommended continuing the original monotherapy in patients whose serum HBV DNA levels were declining and switching to the other drug or combining ETV + TDF/TAF in those with plateauing HBV DNA levels.^6^ However, the quality and certainty of the evidence informing these recommendations are very low. Therefore, prospective multi-center studies are needed to better answer the question of LLV management.

We also analyzed other clinical indicators between groups LLV and CVR at the time point of identifying the occurrence of LLV by performing highly sensitive HBV DNA tests (lower detection limit, 10 IU/mL). Compared with the CVR group, patients who were in the LLV state had higher ALT, AST, HBsAg, AFP levels, and a higher proportion of patients were HBeAg-positive. Therefore, higher ALT, AST, HBsAg, AFP levels, and HBeAg-positivity after more than 12-month antiviral therapy can be used as hints of LLV. Therefore, detecting HBV DNA with highly sensitive HBV DNA tests (lower detection limit, 10 IU/mL) may be further considered in these patients, which is still not prevalent in most hospitals in China.

With the increase in HBV DNA detection sensitivity, LLV has been paid increasing attention. Low-level viremia has been found to be related to the progress of cirrhosis and hepatic carcinogenesis in patients with a cirrhosis background.^11,14^ However, whether patients with LLV are more likely to develop cirrhosis or hepatocellular carcinoma (HCC) in CHB patients without a cirrhosis background is still incompatible,^11,17,18,28^ and some studies found it might be treatment adherence but not LLV that increased the risk of HCC.^22,29^ The optimum level of HBV DNA as the target of nucleos(t)ide analogs (NUCs) therapy is unclear, and more studies urgently need to be done. Of the patients with LLV in our study, 45.1% had DNA levels between 10 IU/mL and 20 IU/mL, and whether it is necessary to take measures to further reduce HBV DNA level to below 10 IU/mL is not clear.

This study has several limitations. First, medication compliance information in our study was difficult to obtain. Poor medication adherence may be a risk factor for LLV, and we cannot accurately assess the influence of adherence. Second, the history of drug resistance in patients receiving other NUC treatments before ETV/TDF monotherapy is unclear. Third, this study has a relatively small sample size.

In conclusion, this study demonstrated that LLV still occurred in a considerable proportion of CHB patients after long-term ETV/TDF treatment. In addition, baseline HBeAg-positivity, a short duration of therapy, and ETV therapy were risk factors for LLV.

## Figures and Tables

**Table 1.** Clinical Characteristics of CHB Patients with CVR or LLV

**Characteristics**	**Total Cohort (n = 158)**	**CVR (n = 107)**		**LLV (n = 51)**	***P***
Age (year)	41.0 (33.0-52.0)	42.0 (33.0-50.0)		40.0 (33.0-54.0)	.958
Male sex (n, %)	89 (56.3)	59 (55.1)		30 (58.8)	.663
Body mass index (kg/m^2^)	23.09 ± 3.20	23.38 ± 3.08		22.95 ± 3.26	.822
Alcohol intake (n, %)	15 (9.5)	9 (8.4)		6 (11.8)	.702
Family history of hepatitis B (n, %)	65 (41.1)	43 (40.2)		22 (43.1)	.725
Family history of HCC (n, %)	23 (14.6)	13 (12.1)		10 (19.6)	.214
Liver cirrhosis (n, %)	28 (17.7)	20 (18.7)		8 (15.7)	.644
Fatty liver disease (n, %)	54 (34.2)	32 (29.9)		22 (43.1)	.101
Diabetes mellitus (n, %)	8 (5.1)	4 (3.7)		4 (7.8)	.476
Hypertension (n, %)	12 (7.6)	8 (7.5)		4 (7.8)	.935
Duration of disease since first diagnosis (year)	16.0 (9.8-20.0)	16.0 (10.0-20.0)		17.0 (6.0-20.0)	.839
Type of drugs (n, %)					.032
ETV	109 (69.0)	68 (63.6)		41 (80.4)	
TDF	49 (31.0)	39 (36.4)		10 (19.6)	
Naïve (n, %)	126 (79.8)	81 (75.7)		45 (88.2)	.067
Duration of therapy (month)	36.0 (21.6-60.0)		36.0 (24.0-66.0)	26.4 (19.0-49.0)	.020
Baseline HBV DNA (log_10_ IU/mL)	6.1 (3.7-7.2)		5.7 (2.8-7.0)	7.0 (4.5-7.5)	.013
Baseline HBeAg positivity (n, %)	105 (66.5)		62 (57.9)	43 (84.3)	.001
Baseline HBsAg (log_10_ IU/mL)	3.49 (3.11-3.99)		3.41 (3.05-3.82)	3.79 (3.29-4.42)	.001
Baseline ALT (U/L)	61.0 (31.0-108.0)		59.0 (26.0-118.0)	64.0 (36.0-100.0)	.307
Baseline AST (U/L)	41.0 (26.0-74.0)		37.0 (24.0-71.0)	45.0 (27.0-85.0)	.171
Baseline GGT (U/L)	25 (15-48)		20 (14-45)	34 (21-62)	.004
Baseline albumin (g/L)	46.0 (43.0-49.0)		47.0 (44.0-49.0)	45.0 (42.8-48.0)	.042
Baseline total bilirubin (μmol/L)	13.6 (10.2-17.8)		13.7 (9.9-18.0)	13.1 (10.4-17.6)	.961
Baseline creatinine (μmol/L)	66±14		66±14	68±13	.227
Baseline AFP (ng/mL)	2.73 (1.93-4.15)		2.38 (1.86-3.60)	3.07 (2.22-5.84)	.018
Baselien PT (s)	11.6 (11.1-12.4)		11.6 (11.2-12.3)	11.4 (10.9-12.6)	.631
Baseline Hb (g/L)	146±18		145±19	149±15	.105
Baseline WBC (×10^9^/L)	5.37±1.55		5.25±1.27	5.61±2.01	.254
Baseline neutrophil (×10^9^/L)	3.11±1.29		3.08±1.06	3.16±1.69	.735
Baseline lymphocyte (×10^9^/L)	1.76±0.85		1.65±0.48	1.97±1.30	.100
Baseline platelet (×10^9^/L)	184.0 (147.0-230.0)		186.0 (150.0-229.0)	181.0 (123.0-232.0)	.455

Data are expressed as the median (interquartile range) or mean ± SD as appropriate or number (percent).

CHB, chronic hepatitis B; CVR, complete virological response; LLV, low-level viremia; HCC, hepatocellular carcinoma; ETV, entecavir; TDF, tenofovir disoproxil fumarate; HBV, hepatitis B virus; HBeAg, hepatitis B e antigen; HBsAg, hepatitis B surface antigen; ALT, alanine aminotransferase; AST, aspartate transaminase; GGT, γ-glutamyltranspeptidase; AFP, alpha fetoprotein; PT, prothrombin time; Hb, hemoglobin; WBC, white blood cells; SD, standard deviation.

**Table 2.** Factors Associated with the Occurrence of LLV

Factors	Univariate Analysis			Multivariate Analysis	
	OR (95% CI)	*P*		OR (95% CI)	*P*
Age (years)	0.947 (0.974-1.028)	.947		-	-
Male	1.162 (0.592-2.283)	.663		-	-
Body mass index (kg/m^2^)	1.042 (0.939-1.157)	.437		-	-
Alcohol intake (n, %)	1.452 (0.487-4.325)	.503		-	-
Family history of hepatitis B (n, %)	1.129 (0.575-2.219)	.725		-	-
Family history of HCC (n, %)	1.764 (0.715-4.348)	.218		-	-
Liver cirrhosis (n, %)	0.809 (0.330-1.986)	.644		-	-
Fatty liver disease (n, %)	1.778 (0.890-3.551)	.103		-	-
Diabetes mellitus (n, %)	2.191 (0.525-9.141)	.282		-	-
Hypertension (n, %)	1.053 (0.302-3.674)	.935		-	-
Duration of disease since first diagnosis (year)	0.996 (0.963-1.029)	.794		-	-
**HBeAg (+)**	**3.901 (1.673-9.097)**	**.003**		**6.479 (2.480-16.922)**	**.000**
**Regimen (ETV)**	**2.351 (1.061-5.210)**	**.035**		**4.742 (1.855-12.118)**	**.001**
Naïve	2.407 (0.922-6.285)	.073		**-**	-
**Duration of therapy (>36 months)**	**0.295 (0.146-0.599)**	**.001**		**0.168 (0.072-0.388)**	**.000**
Baseline HBV DNA (log_10_ IU/mL)	1.254 (1.034-1.520)		.021	**-**	-
Baseline HBsAg (log_10_ IU/mL)	2.371 (1.383-4.064)		.002	**-**	-
Baseline ALT (U/L)	1.001 (0.999-1.003)		.324	**-**	-
Baseline AST (U/L)	1.002 (0.999-1.006)		.200	**-**	-
Baseline GGT (U/L)	1.010 (1.000-1.020)		.054	**-**	-
Baseline albumin (g/L)	0.921 (0.852-0.996)		.039	**-**	-
Baseline total bilirubin (μmol/L)	1.010 (0.991-1.029)		.291	**-**	-
Baseline creatinine (μmol/L)	1.015 (0.991-1.041)		.227	**-**	-
Baseline AFP (ng/mL)	1.018 (0.992-1.045)		.183	**-**	-
Baselien PT (s)	1.079 (0.871-1.337)		.485	**-**	-
Baseline Hb (g/L)	1.015 (0.995-1.036)		.139	**-**	-
Baseline WBC (×10^9^/L)	1.157 (0.932-1.436)		.185	**-**	-
Baseline platelet (×10^9^/L)	0.999 (0.995-1.004)		.716	**-**	-

Bold-face font represents factors that are significant predictors of LLV in multivariate analyses.

LLV, low-level viremia; HCC, hepatocellular carcinoma; HBeAg, hepatitis B e antigen; ETV, entecavir; HBV, hepatitis B virus; HBsAg, hepatitis B surface antigen; ALT, alanine aminotransferase; AST, aspartate transaminase; GGT, γ-glutamyltranspeptidase; AFP, alpha fetoprotein; PT, prothrombin time; Hb, hemoglobin; WBC, white blood cells.

**Table 3.** Baseline Characteristics of the ETV and TDF Treatment Groups After PSM

**Characteristics**	**ETV (n = 36)**	**TDF (n = 36)**	***P***
Age (year)	44.0 (31.5-55.0)	39.0 (33.0-50.5)	.640
Male sex (n, %)	16 (44.4)	18 (50.0)	.637
Body mass index (kg/m^2^)	22.99 (21.02-24.50)	22.90 (20.49-24.14)	.547
Alcohol intake (n, %)	1 (2.8)	5 (13.9)	.199
Family history of hepatitis B (n, %)	20 (55.6)	15 (41.7)	.238
Family history of HCC (n, %)	5 (13.9)	2 (5.6)	.429
Liver cirrhosis (n, %)	6 (16.7)	3 (8.3)	.478
Fatty liver disease (n, %)	11 (30.6)	12 (33.3)	.800
Diabetes mellitus (n, %)	2 (5.6)	1 (2.8)	1.000
Hypertension (n, %)	2 (5.6)	4 (11.1)	.674
Duration of disease since first diagnosis (year)	17.5 (10.0-20.0)	16.5 (5.0-20.0)	.932
Naïve (n, %)	29 (80.6)	28 (77.8)	.772
Duration of therapy (month)	35.5 (20.0-58.8)	40.0 (24.0-74.0)	.262
Baseline HBV DNA (log_10_ IU/mL)	6.0 (3.5-7.3)	6.0 (3.8-7.3)	.964
Baseline HBeAg positivity (n, %)	23 (63.9)	23 (63.9)	1.000
Baseline HBsAg (log_10_ IU/mL)	3.43 (2.99-3.83)	3.59 (3.16-4.05)	.290
Baseline ALT (U/L)	65.5 (28.0-142.5)	72.5 (36.5-119.0)	.906
Baseline AST (U/L)	44.5 (25.5-83.0)	42.0 (28.0-65.5)	.813
Baseline GGT (U/L)	25 (13-58)	19 (12-49)	.773
Baseline albumin (g/L)	45.5 (42.5-49.0)	46.6 (44.5-49.0)	.238
Baseline total bilirubin (μmol/L)	12.8 (10.6-17.4)	14.2 (10.5-19.0)	.517
Baseline creatinine (μmol/L)	61 (53-70)	65 (55-75)	.252
Baseline AFP (ng/mL)	2.91 (2.18-5.16)	2.11 (1.75-3.36)	.056
Baselien PT (s)	11.7 (11.2-12.6)	12.0 (11.6-13.0)	.438
Baseline Hb (g/L)	147 (133-161)	145 (135-157)	.944
Baseline WBC (×10^9^/L)	4.93 (4.31-5.63)	5.55 (4.60-6.09)	.124
Baseline platelet (×10^9^/L)	182.5 (148.5-224.0)	192.0 (138.0-231.0)	.536

Data are expressed as the median (interquartile range) or number (percent).

PSM, propensity score matching; ETV, entecavir; TDF, tenofovir disoproxil fumarate; HCC, hepatocellular carcinoma; HBV, hepatitis B virus; HBeAg, hepatitis B e antigen; HBsAg, hepatitis B surface antigen; ALT, alanine aminotransferase; AST, aspartate transaminase; GGT, γ-glutamyltranspeptidase; AFP, alpha fetoprotein; PT, prothrombin time; Hb, hemoglobin; WBC, white blood cells.

**Table 4.** Laboratory Data of CHB Patients with CVR or LLV at the Time Point of Identifying LLV

**Characteristics**	**Total Cohort (n = 158)**	**CVR (n = 107)**	**LLV (n = 51)**	***P***
HBeAg positivity (n, %)	80 (50.6)	46 (43.0)	34 (66.7)	.005
HBsAg (log_10_ IU/mL)	3.18 (2.80-3.75)	3.12 (2.71-3.56)	3.49 (2.98-3.93)	.033
ALT (U/L)	21.0 (15.0-31.0)	21.0 (15.0-27.0)	28.0 (16.0-46.0)	.008
AST (U/L)	21.0 (18.0-27.0)	20.0 (17.0-26.0)	23.0 (19.0-31.0)	.026
GGT (U/L)	18 (13-29)	18 (12-29)	19 (14-34)	.219
Albumin (g/L)	48.0 (46.0-50.0)	48.0 (46.0-50.0)	47.0 (45.0-49.0)	.072
Total bilirubin (μmol/L)	12.9 (9.6-16.5)	12.8 (9.6-16.5)	13.1 (9.6-18.6)	.929
AFP (ng/mL)	2.73 (1.93-4.15)	2.06 (1.59-2.94)	2.56 (1.99-3.52)	.007
Creatinine (μmol/L)	64±14	64±14	66±14	.334
Platelet (×10^9^/L)	196.0 (159.0-235.0)	196.0 (166.0-235.0)	189.0 (152.0-240.0)	.622

Data are expressed as the median (interquartile range) or mean ± SD as appropriate or number (percent).

CHB, chronic hepatitis B; CVR, complete virological response; LLV, low-level viremia; HBeAg, hepatitis B e antigen; HBsAg, hepatitis B surface antigen; ALT, alanine aminotransferase; AST, aspartate transaminase; GGT, γ-glutamyltranspeptidase; AFP, alpha fetoprotein; SD, standard deviation.
